# Low-cost Fabrication of Tunable Band Gap Composite Indium and Gallium Nitrides

**DOI:** 10.1038/s41598-019-38882-3

**Published:** 2019-02-19

**Authors:** Andrew McInnes, Jagdeep S. Sagu, Diana Mehta, K. G. U. Wijayantha

**Affiliations:** Energy Research Laboratory, Department of Chemistry, Loughborough University, Loughborough, LE11 3TU UK

## Abstract

III-nitride materials have been linked with a vast number of exciting applications from power electronics to solar cells. Herein, polycrystalline InN, GaN and systematically controlled In_x_Ga_1−x_N composite thin films are fabricated on FTO glass by a facile, low-cost and scalable aerosol assisted chemical vapor deposition technique. Variation of the indium content in the composite films leads to a dramatic shift in the optical absorbance properties, which correlates with the band edges shifting between those of GaN to InN. Moreover, the photoelectrochemical properties are shown to vary with indium content, with the 50% indium composite having an external quantum efficiency of around 8%. Whilst the overall photocurrent is found to be low, the photocurrent stability is shown to be excellent, with little degradation seen over 1 hour. These findings demonstrate a new and low-cost method for fabricating polycrystalline III-nitrides, which have a range of interesting properties that are highly sought after for many applications.

## Introduction

III-nitride semiconductors, particularly based on indium and gallium, have long been recognized as some of the most promising materials for a wide range of future electronic and optoelectronic applications^1^. GaN is the most studied III-nitride material and its superior chemical and physical stability makes it an ideal semiconductor for use in more harsh environments^2–4^. Due to its excellent thermal stability, coupled with the ability to form a high mobility two dimensional electron gas (2DEG), GaN is regarded as the ideal candidate material to replace silicon in many high-frequency devices^5–8^. In addition to the excellent electronic properties, the wide band gap of GaN means it also forms the basis of the incredibly successful violet laser diodes (LDs) and blue and green light emitting diodes (LEDs), achieved by alloying with indium, which are found at the heart of most modern optical data storage technologies^9–11^.

Comparatively, InN has been less well studied, but holds a significant promise as a future electronics material. The material has very attractive electron transport characteristics and at a relatively small doping density, the mobility can be as high as 12,000 cm^2^/Vs. Its effective mass of the lowest valley is about 0.04m_0_, such that it would be ideal for high-speed electronic devices^12,13^. Furthermore, its small but direct band gap, between 0.6–0.7 eV, makes InN ideal for use in photovoltaics, LEDs and photoelectrochemical devices^14,15^. Due to its strong intrinsic surface charge accumulation properties (i.e. surface charge of the order of 10^13^ cm^−2^) together with excellent chemical stability, InN has been investigated as a promising active material in chemical and biosensor applications^16^.

Fabrication of single crystals of InN, GaN and their alloys have often been achieved using highly controlled physical deposition methods such as Molecular Beam Epitaxy^17^, Metal-Organic Vapor Phase Epitaxy^18^, High-Pressure Chemical Vapor Deposition^19^, Atomic Layer Deposition^20^ and Reactive Sputtering^21^, many of which are regarded as being expensive and difficult to deposit over a large area. Moreover, careful selection of substrates is often required as a large mismatch in the lattice constants and thermal expansion coefficients exist for many common substrates^22^. In contrast, polycrystalline InN and GaN thin films have the potential to be utilized for a large number of applications^23–28^. For example, despite the high density of dislocations generally associated with polycrystalline InN and GaN, highly efficient LEDs have still been achieved^29^. They can also be deposited over a very large area on a variety of substrates. The less stringent deposition criteria required for deposition of polycrystalline films have offered new avenues to form alloyed In_x_Ga_1−x_N materials, targeting a variety of new and attractive applications such as highly efficient graded bandgap solar cells, highly efficient LEDs, breaking through traditional limitations in thermoelectrics (by using the exceptionally strong internal polarization fields present in III-Nitride materials), power electronics and photoelectrochemical devices.

Literature suggests that alloy films with high indium contents have typically been difficult to obtain, due in part to a solid phase miscibility gap that exists between the InN and GaN phases^30^. Additionally, the lattice constants of InN, GaN and their alloys differ significantly, often leading to the formation of materials with poor crystalline quality^31^. However, polycrystalline III-nitrides could potentially offer means to reduce the crystallographic strain and improve alloy formation, if a low-cost and scalable deposition method is identified. One of the most practical methods for the preparation of polycrystalline thin films on a large scale is chemical vapor deposition (CVD)^32,33^. Moreover, Aerosol Assisted Chemical Vapor Deposition (AACVD), a variant of the CVD technique which utilizes aerosol droplets to transfer precursors to the heated substrate, is one of the best methods for fabricating mesoporous polycrystalline thin films as requirements on precursor volatility and thermal stability are completely negated^33–36^. The method is simple, low-cost and easily scalable yet it has mainly been used to make single and multi-component metal oxides thin films as well as oxide based composites and solid-solutions. As a thin film preparation technique, AACVD offers numerous advantages, as modification of the technique, through varying the solvent, carrier gas flow rate, precursor concentration and aerosol droplet size, allows control over the thin film morphology and particle size which are often difficult to achieve by other methods^37–39^.

In this study^40^, we have demonstrated that polycrystalline InN, GaN and systematically controlled In_x_Ga_1−x_N composite thin films can be successfully fabricated on fluorine-doped tin oxide coated glass substrates by AACVD. Variation in the indium content of polycrystalline nitride composite thin films was found to lead to a dramatic shift in the optical absorbance. Importantly, the band edges of the composites shift between that of GaN to that of InN, which is a fundamental objective of many theoretical studies reported in the literature to date. Furthermore, we have observed a striking correlation in the trend between the photocurrent onset potential of our polycrystalline InN, GaN and composites and the band edges determined by Mott-Schottky analysis. It is also important to note that although it was difficult to conclusively prove the formation of In_x_Ga_1−x_N alloys by high resolution X-ray diffraction measurements, due to the polycrystalline nature of the thin films, the optical and electrical measurements have indicated the formation of alloys (for example, a significant modification to the band gap could be achieved through changes to the indium content of the thin films, realized by modulation the ratio of precursor flow rates in the thin film fabrication process. A calibration plot for the composition was created with respect to the flow rates for facile tuning of the band gap). However, in order to avoid any doubts, we have opted to call them ‘composite’ thin films in this report.

Photocurrent density is relatively low which is attributed to the high packing density of the thin films and their low surface area, but the stability of the composite nitride film (which corresponds 11% indium) and InN film were found to be excellent, with little in the way of photocurrent degradation during the 1 hour measurement time. These findings will have an immediate and significant impact on the current efforts on low-cost fabrication of bandgap tunable polycrystalline III-nitride semiconductor thin films for wide range of applications.

## Methods

InN, GaN and In_x_Ga_1−x_N composite thin films were fabricated by AACVD using separate indium and gallium precursor solutions. Gallium chloride and indium chloride (Sigma Aldrich, 99.999%) were dissolved in acetonitrile to produce 0.1 M solutions respectively. Commercially available FTO (TEC 8 Pilkington, 8 Ω/sq.) was used as the substrate for the deposition. Substrates were cut, using a diamond edged blade, by hand (1 cm × 7 cm) and ultrasonically cleaned in deionized water, acetone, propan-2-ol and then stored in ethanol. Substrate slides were removed from the ethanol and dried just prior to the nitride thin film fabrication. Substrates were placed in a glass chamber of a tube furnace and heated under nitrogen for 15 minutes at 600 °C prior to each deposition.

For a typical deposition, round bottom flasks containing the indium and gallium precursors were placed over the piezoelectric modulators of two ultrasonic humidifiers (Vicks ultrasonic humidifier, model number: VH5000AE1) to generate the precursor aerosols. Nitrogen was used as the carrier gas to transfer the aerosol into the mixing chamber. Flow rates were varied for each precursor chamber between 59 and 470 cm^3^min^−1^ in defined amounts such that the combined total flow rate of both precursor aerosols was maintained at 529 cm^3^min^−1^. Anhydrous ammonia gas (BOC gases Ltd.) was added into the mixing chamber as a reactive gas, at a flow rate of 862 cm^3^min^−1^. The high flow rate of ammonia assisted in promoting smaller droplets into the decomposition chamber whilst also ensuring an excess of ammonia to create nitride thin films. The aerosol droplets entering the decomposition chamber land on the heated substrate where they decompose forming nitride thin films. The composition of the thin film is dependent on the ratio of the flow rates of the indium and gallium precursors. The deposition was continued for 1 hour to produce films of suitable thickness, approximately 2 μm. At the end of the deposition the films were allowed to slowly cool down to room temperature under a flow of nitrogen. Figure S1 in the supporting information, shows a schematic diagram of the deposition method. Control experiments conducted by subjecting bare FTO substrates to identical heating cycles (without the precursor flow) indicated that substrates did not undergo deformation under the deposition conditions.

The phase and crystallinity of the films were characterized using a Bruker AXS Advance X-ray diffractometer (XRD) with primary monochromatic high intensity Cu Kα (λ = 1.541 Å) radiation. The current-voltage (*J–V*) characteristics and C-V characteristics of the thin films were conducted using a Galvanostat/Potentiostat (Eco Chemie micro-Autolab type III), under illumination of simulated sunlight by an AM 1.5 Class A solar simulator (Solar Light 16S – 300 solar simulator), at 100 mW cm^−2^ light intensity, calibrated by a silicon pyranometer (Solar Light co., PMA2144 Class II). The films were measured in a standard three-electrode configuration in a quartz cell using a platinum wire counter-electrode and Ag|AgCl|3 M KCl reference electrode. The electrolyte was 1 M sulfuric acid and the scan rate was maintained at 10 mV s^−1^. The internal quantum efficiency (IQE) was obtained by measuring the incident photon flux using a 75 W xenon lamp connected to a monochromator (TMc300, Bentham Instruments Ltd., Berkshire, UK). The light was calibrated using a silicon diode. Photocurrent spectra were measured at 1.5 V vs Ag/AgCl, in the same 3 electrode cell set up and photocurrent measurements, using an analogue potentiostat (Whistonbrook Technologies Ltd.). Readings were taken at 5 nm intervals whilst the monochromated light scanned from 300 to 700 nm. The absorbance and reflectance spectra of the FTO glass substrates were used to correct the data to yield internal quantum efficiency values. Optical absorbance measurements were carried out on a Lambda 35 Perkin-Elmer UV/Vis Spectrometer. The surface morphology was studied using a Leo 1530 VP field emission gun scanning electron microscope (FEG-SEM) at an accelerating voltage of 5 kV and a working distance of 5 mm. Energy-dispersive X-ray (EDX) spectroscopy was carried out in order to determine the In/Ga ratio. X-ray photoelectron spectroscopy (XPS) was recorded using a Thermo Scientific K-Alpha XPS spectrometer operated with an unmonochromated Al Kα X-ray source (1486.6 eV).

## Results and Discussion

Group III nitride thin films, specifically InN and GaN, have proven extremely difficult to grow under the same conditions on practical substrates, as they typically require significantly different growth temperatures: 500 °C or below for InN and above 750 °C for GaN^41^. Moreover, the successful formation of an In_x_Ga_1−x_N alloy phase, which is highly sought after in the fields such as solar cells, LEDs and thermoelectrics, is critically dependent on the ability to grow both InN and GaN under the same conditions. Aerosol Assisted Chemical Vapor Deposition was used to successfully deposit polycrystalline InN, GaN and composite thin films at 600 °C on fluorine-doped tin oxide coated glass.

**Figure 1:**
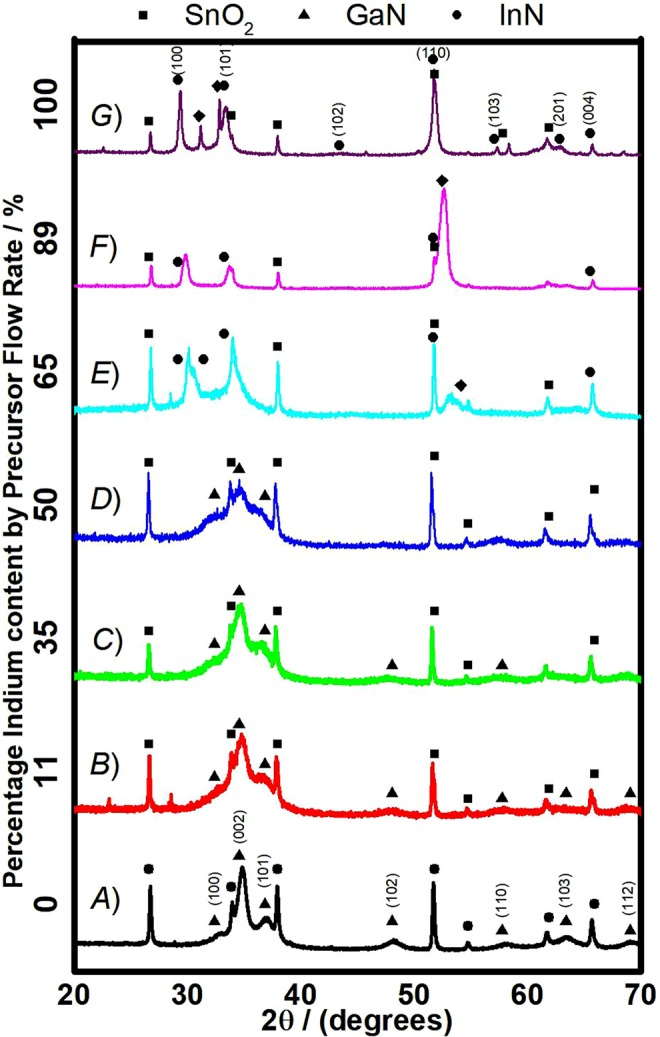
XRD patterns for thin films with various percentages of indium precursor determined by the deposition flow rate including (**A**) 0%/GaN (Black); (**B**) 11% (Red); (**C**) 35% (Green); (**D**) 50% (Blue); (**E**) 65% (Cyan); (**F**) 89% (Pink) and (**G**) 100%/InN (Purple).

X-Ray Diffraction was used to characterize the phase and crystallinity of the nitride thin films. Diffraction patterns for the as deposited thin films are shown in Fig. 1. For samples fabricated with 100% indium precursor, InN reflections corresponding to the (1 0 0), (0 0 2), (1 0 1), (1 0 2), (1 1 0), (1 0 3), (2 0 1) and the (0 0 4) crystal phases were observed (ICDD 01-070-2547), which is a hexagonal structure. Several reflections can also be assigned to the formation of In_2_O_3_, specifically the (1 0 4) and the (1 1 0) crystal phases (ICDD 00-022-0336). All other reflections observed can be attributed to the FTO substrate and include reflections for the (1 1 0), (1 0 1), (2 0 0), (2 1 1), (0 0 2) and (3 1 0) crystal phases respectively (ICDD 00-041-1445). Interestingly no metallic indium phases are observed in the InN thin films. Indium nitride is known to have a low decomposition temperature, around 550 °C, which usually leads to the formation of indium droplets and phase separation, particularly at the surface^42^. Previous studies have suggested that the surface segregation can be reduced by growth in a nitrogen-rich environment, which is similar to the conditions under which the thin films are grown by AACVD in our case^43^. Furthermore, metallic indium readily bonds with oxygen and as it is not possible to guarantee complete oxygen removal from the apparatus we used, it is possible that any indium droplets that do form may undergo rapid oxidation, forming the In_2_O_3_ phases that we observed^44^. In_2_O_3_ has a band gap of 3.6 eV, so is not likely to affect the light absorbing properties of the InN material in these thin films significantly. Furthermore it has not been observed in any of the low In content composite thin films, which would have similar optical absorbing properties, so is not believed to contribute significantly to the observed optical properties. Despite the fact that oxides are highly defective and the defect related transitions could happen in the range of 1.7–2.3 eV, synthesis of pure In_2_O_3_ thin films by AACVD did not result in band gaps within this range, therefore, the reduction in the gap at higher In content is not likely to be due to this.

GaN thin films were also fabricated, corresponding to the 0% indium precursor flow rate samples. For these thin films, GaN reflections corresponding to the (1 0 0), (0 0 2), (1 0 1), (1 0 2), (1 1 0), (1 0 3) and the (1 1 2) were all observed (ICDD 01-076-0703), which is also a hexagonal structure. Again, all remaining reflections observed were attributed to the FTO substrate. As expected, in accordance with the literature, the GaN reflections are broad indicating a greater number of defects and a poorer crystallinity, potentially caused by the lower growth temperature^45,46^. Additionally some of the crystal reflections may have a slight shift compared to their expected positions, which again indicates expansion or contraction within the crystal lattice caused by defects.

In order to investigate the ability for alloy thin film formation via AACVD, a dual precursor apparatus was used and the ratio of the precursor flow rates was systematically varied, leading to a variation in the composition of the resulting thin films. Thin films fabricated with less than 50% indium precursor showed reflections predominantly for GaN crystal phases, whilst over 50% showed more InN character. As the indium precursor percentage increased, from pure GaN up to 50% flow rate, the reflections were seen to become increasingly broader, indicating a decrease in the crystallinity of the thin films. This broadening has been extensively reported in the literature as scattering which may be due to the formation of defects caused by incorporation of indium into the GaN crystal lattice^47^.

For thin films fabricated with more than 50% indium precursor, the reflections observed were exclusively for InN crystal phases, again with broad features becoming more refined with increasing indium input. Interestingly all the thin films showed only a single crystal structure, either GaN or InN, and mixtures of both were not observed. This indicates that a crystalline solid solution has not been formed. Furthermore, for the thin films fabricated with 89% and 65% indium precursor, a shift in the (1 0 0) reflection to higher angles may also be observed. The peak is seen to become broader indicating decreasing crystallinity, particularly for the 65% thin film, however the shift is certainly noteworthy. A shift in peak angle like this could indicate a reduction in the crystal lattice parameter, which may be indicative of the incorporation of gallium into the InN crystal lattice, as Ga has a smaller ionic radius.

**Figure 2:**
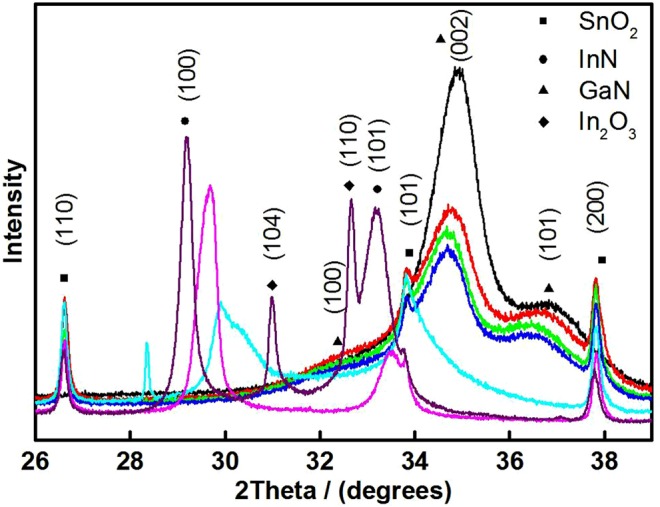
High Resolution ω/2θ pattern for nitride thin films with various indium contents including GaN/0% (Black), 11% (Red), 35% (Green), 50% (Blue), 65% (Cyan), 89% (Pink) and InN/100% (Purple). Reflections for SnO_2_ (Squares), InN (Circles), GaN (Triangles) and In_2_O_3_ (Diamonds) are highlighted.

To investigate the potential alloy formation further, high resolution 2θ scans were carried out over the 25–40° range on the composite thin films as shown in Fig. 2. Typically, one is able to discern if an alloy phase has been successfully formed by looking at the shift in the GaN (0 0 2) reflection to smaller angles, indicating the expansion of the crystal lattice by incorporation of the larger In ions^42^. In polycrystalline thin films, spotting a shift in this way is particularly challenging due to the presence of multiple crystal phases. From the ω/2θ patterns it is not clear whether there is a shift in the (0 0 2) reflection, primarily because of the extremely broad peaks and also the overlap between different reflections. The shift in the (1 0 0) InN reflection to larger angles is quite distinct however, for the 65 and 89% indium precursor fabricated films.

Unfortunately, the XRD results cannot conclusively prove the formation of an In_x_Ga_1−x_N alloy crystal phase. A solid solution, however, would be expected to show reflections for both InN and GaN crystal phases, which is not seen for our polycrystalline AACVD thin films. The shift in the InN (1 0 0) reflection to larger angles and the broad reflections observed for the low indium thin films are all suggestive of a level of dopant incorporation into a primary crystal lattice (GaN for low indium and InN for high indium). The difficulties in forming high indium content In_x_Ga_1−x_N alloys have been well reported in the literature and are primarily due to a solid-phase miscibility gap that occurs between InN and GaN^30,48–50^. However, In_x_Ga_1−x_N phases with high indium contents have been produced in materials with a highly nanostructured morphology, such as nanowires^50^. With the lack of conclusive proof of alloy formation, we have opted to refer to these polycrystalline thin films as “composites”, which may comprise InN, GaN and an In_x_Ga_1−x_N alloy phase.

**Figure 3:**
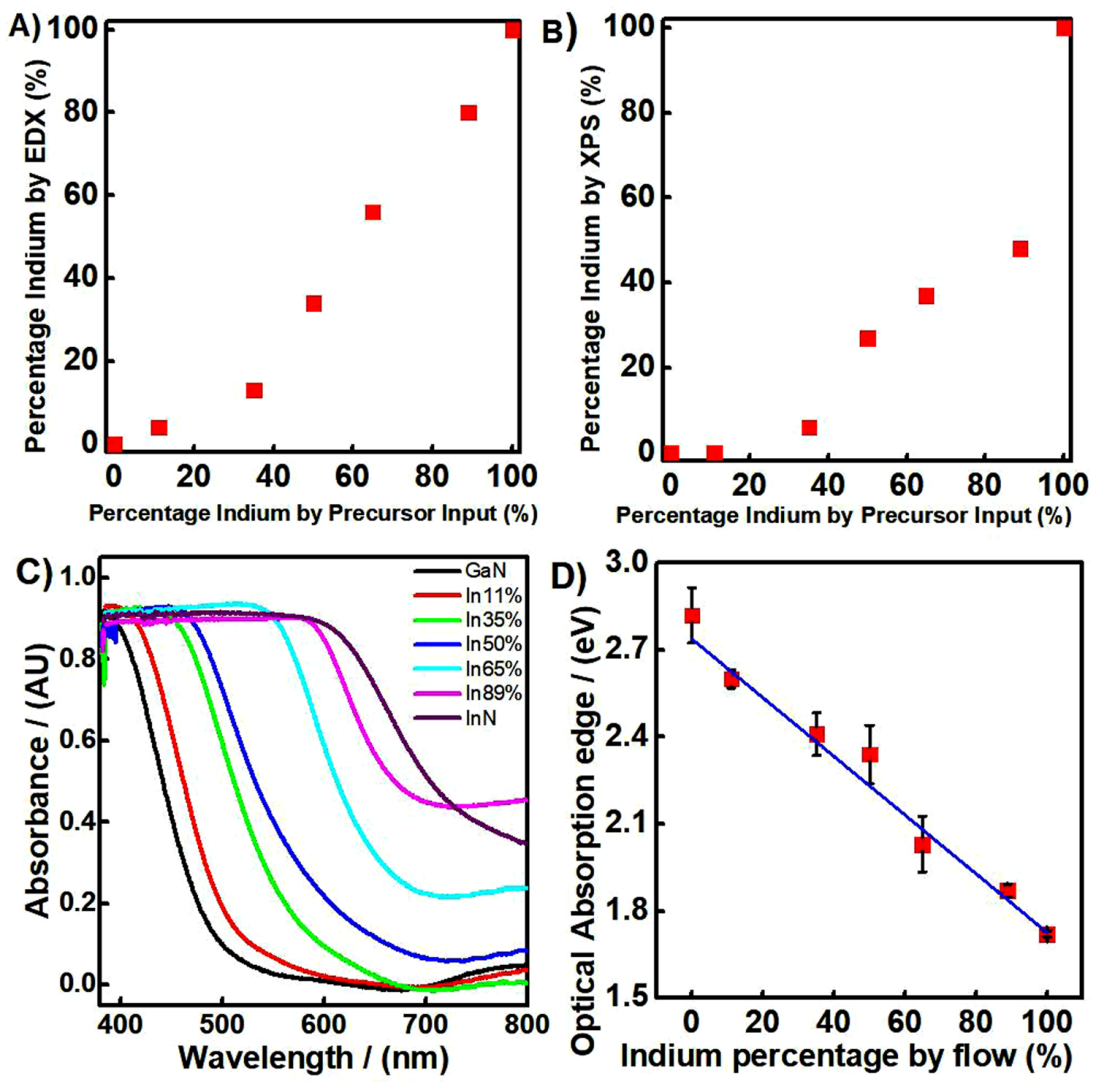
(**A**) Comparison of the percentage indium content observed in EDX with the indium precursor input ratio; (**B**) Comparison of the percentage indium content observed in XPS with the precursor input ratio; (**C**) Diffuse UV-Visible Absorbance Spectra for the range of nitride thin films; (**D**) Calibration plot for the indium input ratio and the optical absorption edge of the resulting thin films.

In order to quantify the actual In:Ga ratio present in the polycrystalline composite thin films, analysis by Energy Dispersive X-Ray and X-Ray Photoelectron Spectroscopy was carried out. XPS has a penetration depth of only 12 nm compared to EDX, which penetrates by up to 3 μm. This allows EDX to provide an average for the composition of the bulk of the thin film, whilst XPS provides the elemental composition mainly at the surface. The elemental In:Ga ratio from both techniques has been plotted against the precursor flow rate ratio, Fig. 3A,B, with the comprehensive data outlined in the Supporting Information Figs S2 and S3. XPS highlights that the surface composition is relatively poor in indium compared to the bulk, which may indicate a change in composition at the surface compared to the bulk. Additionally, the EDX bulk elemental analysis shows that the general percentage of indium is lower than the percentage in the precursor input. This may indicate that the indium precursor does not decompose as efficiently under the deposition conditions, lowering the indium content in the film. It may also be due to the greater evaporation of metallic indium under the deposition conditions compared to gallium, as we know indium metal can be formed under the deposition conditions and it also has a higher vapor pressure compared to gallium^51^.

Diffuse UV Visible absorbance measurements were carried out on the InN, GaN and the composite thin films to elucidate their optical properties. Tauc plots were constructed to allow more accurate estimation of the band gap energy. Thin films of InN fabricated by AACVD were found to have a band gap value of approximately 1.7 eV. This lies well within the reported band gap range for InN, which varies continuously over the ~0.7 to 2 eV range^9^. The cause for the wide range of band gaps for InN has been discussed at length in the literature. The widening of the band gap, which is approximately 0.7 eV, is in part caused by a high carrier concentration, which forces the Fermi level to reside within the conduction band. Upon photoexcitation, electrons must promote to energy levels above the Fermi level, due to the Pauli Exclusion Principle, which requires photons of higher energy. This is known as the Moss-Burstein Effect^52^. The magnitude of this effect has been questioned, however, and is therefore unlikely to contribute to the entire band gap shift. For example, the band gap widening due to Burstein-Moss shift associated with different doping levels and/or strains has only shown the increase of InN band gap up to about 1.7 eV^53^. Our observation of the systematic shift of the optical band gap from 1.7 to 2.8 eV is very unlikely to be due to the Burstein-Moss shift. Oxygen inclusion has also been reported to lead to an increase in the band gap of polycrystalline InN, leading to similar values to those in the AACVD InN thin films^54^. Finally, variations in the stoichiometry, potentially due to the growth temperature or excess nitrogen, have also been previously connected with notable changes in the band gap, particularly for polycrystalline InN^44,55^. In reality, for the polycrystalline InN thin films, it is reasonable to assume that all of these mechanisms could play some part in the widening of the band gap.

In contrast to InN, GaN has a well reported band gap of 3.4 eV^56^. Polycrystalline GaN thin films fabricated by AACVD, however, have a smaller than expected band gap of 2.82 eV. Previously reported literature points to the fact that the lattice parameters of GaN deviate significantly from those of the substrates on which it is grown, a scenario that may cause significant crystal strain and defect formation^57^. Tensile strain has also been reported to cause significant alteration to a semiconductor band structure and decrease the band gap^58^. It is therefore plausible to suggest that the reduction in the band gap of the polycrystalline GaN thin films could be due to extensive defects and increased strain within the crystal lattice. The presence of a defect rich crystal lattice in our films is supported by the XRD and HR XRD, reported in Figs 1 and 2 respectively, which show very broad peaks, indicative of a highly defective crystal structure^47^.

Variation of the indium content in In_x_Ga_1−x_N materials should allow complete tunability of the band gap, as has been shown previously^50^. Indeed, variation in the indium content of our polycrystalline composite thin films was found to lead to a dramatic change in the diffuse absorbance spectra. The optical absorption onset clearly shifts to longer wavelengths with the increase in indium content. The band gap values can be directly correlated with the percentage indium input to produce a calibration plot, Fig. 3D. The corresponding plot allows one to select the indium input flow rate required to achieve a desired optical absorption edge in the resulting thin film after deposition, something that is incredibly useful in tuning thin films with different band gaps. The error bars are calculated through the standard deviation of multiple samples fabricated at the same nominal flow rates and error itself is due to small variations in the aerosol flux produced by the ultrasonic humidifiers. The accurate selection of specific band gap thin films in this way is highly desirable for many optoelectronic applications and is readily achievable by the AACVD technique^59–61^. One can improve the precision further by using a TSI collision atomizer or an ultrasonic nozzle, instead of the ultrasonic humidifier.

**Figure 4:**
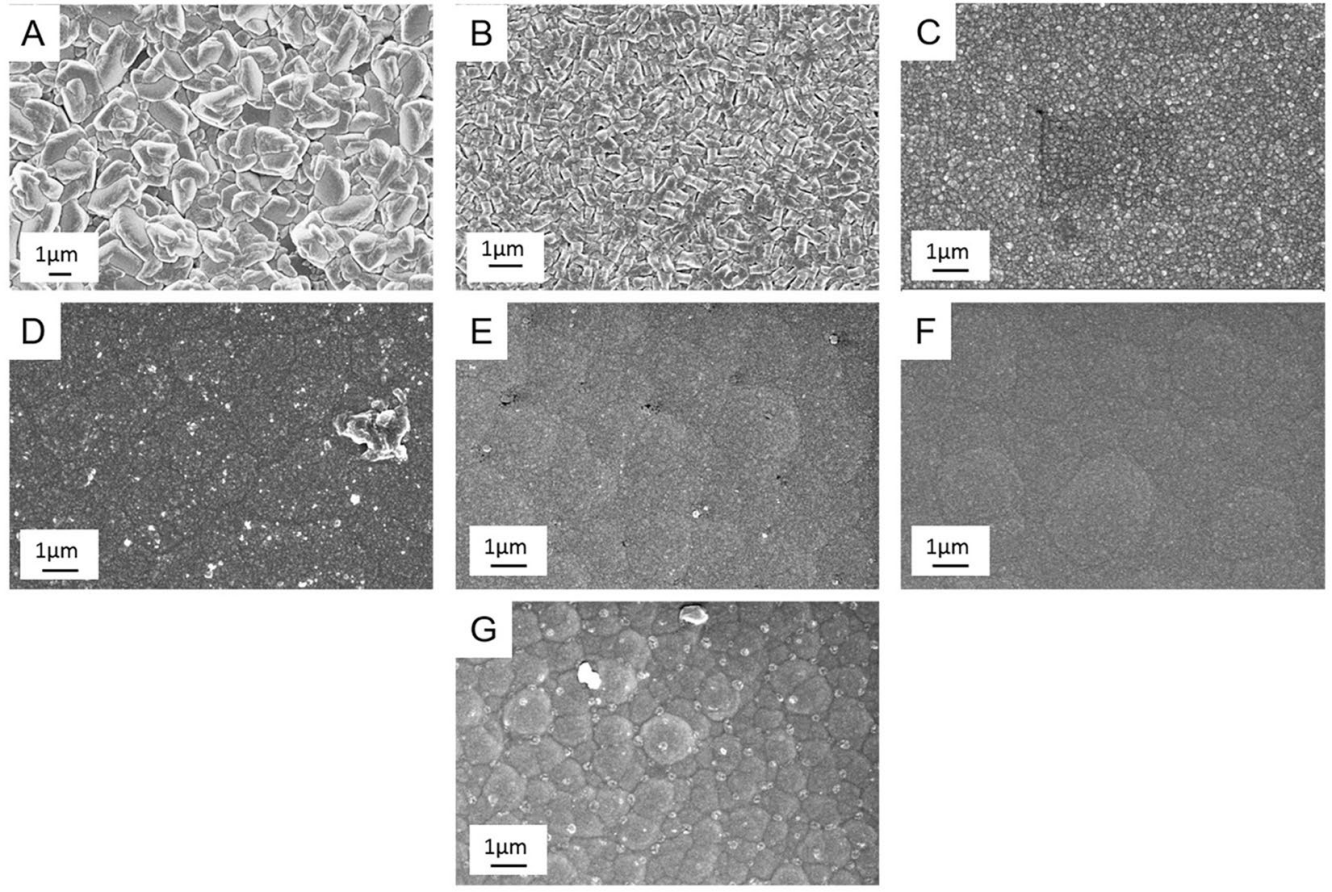
Scanning Electron Micrographs for nitride thin films with percentage indium precursor flow rates of 100%/InN (**A**), 89% (**B**), 65% (**C**), 50% (**D**), 35% (**E**), 11% (**F**) and 0%/GaN (**G**).

The morphology of the InN, GaN and composite thin films was analyzed through scanning electron microscopy. InN thin films were found to have a granular structure with large, sintered features and Fig. 4A describes the surface topography of a representative film. Conversely GaN thin films, Fig. 4G, were found to be very smooth with a rippled surface. The morphology of composite thin films, Fig. 4B–F, was found to vary with the indium content. Thin films fabricated with an 89% indium precursor flow rate have granular features, similar to that of the pure InN films, but smaller in size. Similarly, thin films fabricated with 65% indium precursor flow have a granular structure with very small features. Composite thin films fabricated with 50% or less indium precursor flow rates are found to have much smoother morphologies, with a similar although less defined rippled structure to the GaN thin films. The rough trends in the morphology match well with the trends seen in the XRD and the apparent crystal structures that dominate in these composite thin films.

To determine the growth rate of the AACVD III-nitride thin films, composite thin films, with a pre-selected band gap of approximately 2 eV, were fabricated for times of between 20 and 60 minutes. Cross-sectional SEM was then used to determine the thickness of each composite nitride layer, see Supporting Information Figs S4–S7. Film thicknesses were found to be approximately 2.6, 2.4, 2.0, 1.3 and 0.9 μm for deposition times of 60, 50, 40, 30 and 20 minutes, respectively. From this and the associated error, a growth rate of 50 nm per minute was estimated. In addition, correlation of the band gap with the thickness highlighted that there was no meaningful variation in the band gap with thickness, suggesting a uniform growth of this composition over time.

**Figure 5:**
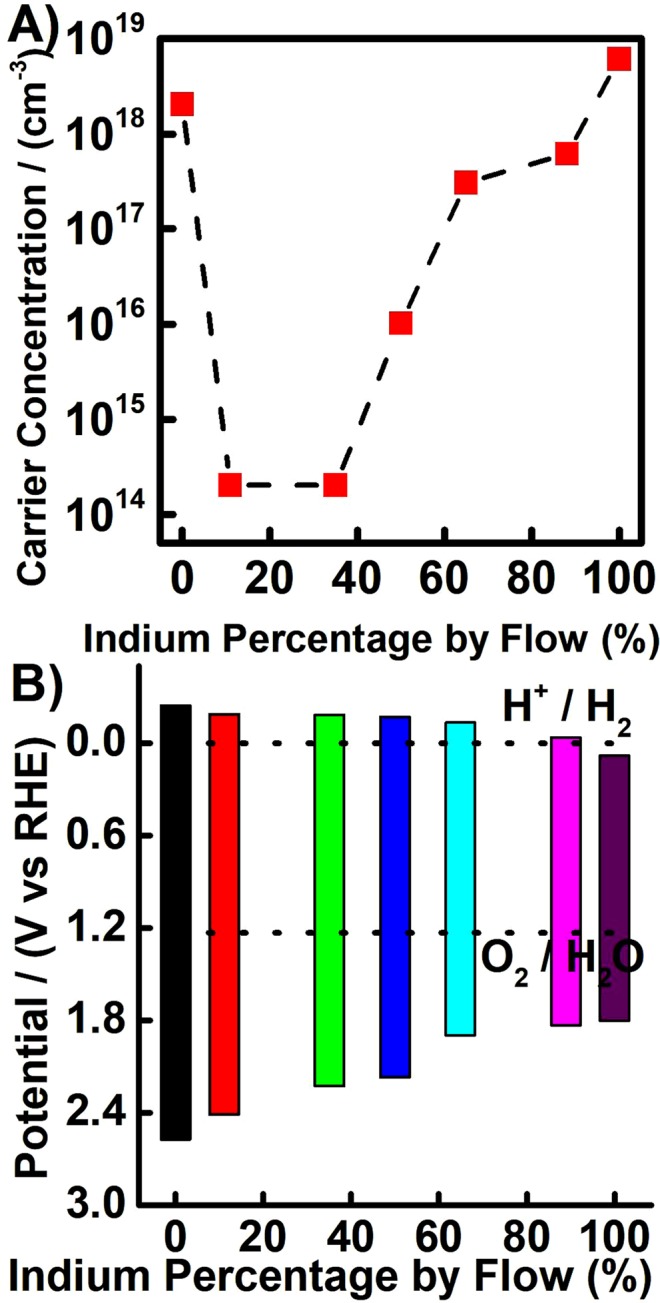
(**A**) Carrier concentration determined by Mott Schottky Analysis for various composite nitride thin films; (**B**) Band edges of InN, GaN and composite thin films relative to the RHE reference scale as measured by Mott-Schottky.

One of the main reasons that InN and GaN are of such interest is because of the ability to modify their electronic properties by alloying them. Herein, Mott-Schottky analysis was used to study the electronic properties of InN, GaN and the composite thin films, a technique which has been previously used in the literature^62–65^. The electrical double layer capacitance was measured over a range of applied voltages and the slope of the resulting plot allows one to estimate the carrier concentration of the semiconductor photoelectrodes. Figure 5A shows the carrier concentration with respect to the indium input ratio. Indium nitride thin films were found to have a high carrier concentration, in the region of 6 × 10^18^ cm^−3^. This correlates well with the literature values in which the high unintentional doping of InN has been reported extensively^66^. InN has also been shown to possess a high surface electron concentration, which coupled with the high carrier concentration, often makes the thin films conducting^67^. The high carrier concentration demonstrated for InN thin films may be due to oxygen incorporation, which has been shown to be an effective n-type donor than alters the band structure^68^. It is also frequently observed that the incorporation of hydrogen, from the decomposing ammonia, into the InN structure can lead to enhanced conductivity^69^. To explore the conductivity of the polycrystalline InN, AACVD was used to fabricate very thin films of InN on plain glass. Films deposited for only 90 seconds were found to be conducting, with the lowest sheet resistance value of 497 Ω/□ and a transparency of 70% at 400 nm, giving a resistivity of 1.99 × 10^−2^ Ω.cm. Other materials with similar resistivity include ITO fabricated by different methods^70,71^. Our brief experiment, without optimization, suggested that conducting InN thin films could be fabricated using AACVD.

GaN thin films were also found to have a high carrier concentration. This could be due to extensive formation of defects within the GaN crystal structure. The resulting thin films were not, however, conducting when deposited on plain glass. The composite thin films were found to have lower carrier concentrations than both the InN and the GaN, which is undoubtedly linked to the complex crystal structure formed with the mixture of the two primary phases.

Mott-Schottky analysis was also used to estimate the flat band potential for the InN, GaN and composite thin films. The flat band potential, in highly n-type doped semiconductor, can be assumed to be equal to the position of the conduction band, which when combined with the optical band gap, allows one to determine the band edge positions. Figure 5B shows the band edge positions with respect to the indium percentage by precursor flow rate for the composite thin films. The band edges for III-nitrides have been reported previously and those measured for the polycrystalline nitride composites are in good agreement with the literature^72,73^. It is clearly seen that the band edges of the composites shift between those of GaN, at approximately −0.24 and 2.57 V vs RHE, to those of InN, at 0.08 to 1.80 V vs RHE respectively. This also indicates that all of the fabricated nitride thin films, with the exception of InN, have band edges that straddled both water redox potentials, a desirable and highly sought property for photoelectrochemical applications such as water splitting and CO_2_ reduction. The correlation between the trend in the band edges determined for the polycrystalline composite nitride thin films with the other literature for In_x_Ga_1−x_N is evidence to suggest the formation of an alloy phase.

**Figure 6:**
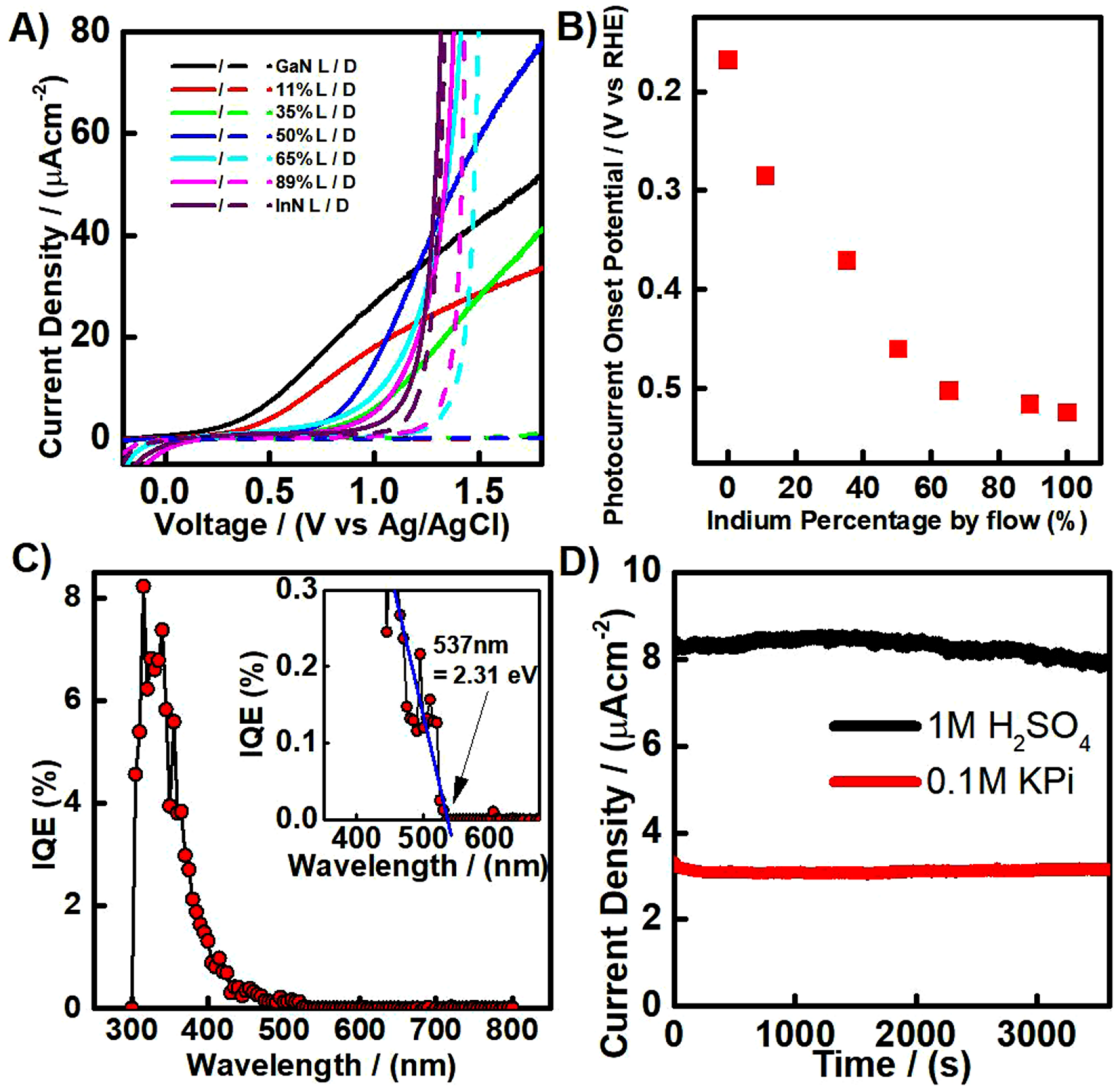
(**A**) Current – Voltage curves measured in 1 M sulfuric acid for composite thin film nitrides with 0%/GaN (Black), 11% (Red), 35% (Green), 50% (Blue), 65% (Cyan), 89% (Pink) and 100%/InN (Purple) indium percentage by precursor flow rate; (**B**) Photocurrent onset potential vs indium content; (**C**) Internal Quantum Efficiency plot for the 50% indium composite thin film measured with an applied voltage of 1.5 V vs Ag/AgCl, the optical photocurrent onset is highlighted through a zoomed in plot (inset); (**D**) Stability curves for 11% composite thin films in 1 M H_2_SO_4_ (Black) and pH 7.4 buffered 0.1 M KPi (Red) electrolytes respectively.

In an effort to determine the potential for polycrystalline nitride thin films to be used as light harvesting layers/films, photoelectrochemical studies were carried out by recording current density – voltage characteristics in 1 M sulfuric acid electrolyte, as shown in Fig. 6A. The L and D curves represent light and dark measurements, respectively.

Photogenerated current, in this case, is caused by the oxygen evolution taking place at the surface of each photoanode. It is clearly observed that the photocurrent density varies significantly with the composition of the thin films. Low indium content composite thin films, up to 50% indium content by precursor flow, were found to be photoactive over a wide potential window, whilst high indium content composites showed much lower photoactivity.

Careful interrogation of the photocurrent onset potential can reveal complimentary information about the band edge positions measured by Mott-Schottky, as shown in Fig. 6B. The photocurrent onset, in an ideal semiconductor photoelectrode, would theoretically be equivalent to the flat band potential. In reality, as the photocurrent is caused by the oxygen evolution reaction, which has sluggish kinetics, recombination of photogenerated charge carriers leads to a shift in the photocurrent onset potential^74^. This phenomena is well known and widely reported in the literature^75,76^. Nevertheless, after discounting the shift in actual potential value, one can observe the striking correlation in the trend between the photocurrent onset potential and indium percentage with the band edges determined by Mott-Schottky. Determination of this trend by two separate methods strengthens this result.

It would be reasonable to expect that with a decrease in the band gap, an increase in the photocurrent would be observed due to an increased amount of visible light absorption. Indeed, the highest photocurrent observed was achieved with the 50% indium composite thin film, which partially meets this expectation. Unfortunately, the thin films prepared by further increases in indium content showed significantly lower photocurrent. Photocurrent density is not simply due to the band gap excitation, as surface kinetics, recombination, crystallinity, thickness, carrier concentration and porosity all play a part in the net photocurrent response^74^. This may explain why the photocurrent for the 11% and 35% thin films was lower than that for GaN, which has a wider band gap than both. Both of these composites showed lower carrier concentrations compared to the GaN. Additionally, differences in porosity can dramatically affect the electroactive surface area of the thin films (Fig. S5 provides an insight to difference in porosity of the series of III-nitrides films made in this study).

In order to investigate the quantum efficiency of nitride thin films, absorbed photon to electron conversion efficiency analysis was carried out on the 50% composite thin film, Fig. 6C. The peak efficiency was found to be approximately 8% at around 315 nm, with a rapid decrease in efficiency for longer wavelengths. Close inspection of the wavelength at which the photocurrent starts yields the optical onset, which in turn reveals the optical band gap, shown inset in Fig. 6C. The optical onset was observed at 535 nm, which corresponds to an optical band gap of 2.32 eV. When taking account of experimental errors, this matches almost exactly with the value obtained from diffuse UV Visible Absorbance measurements. This indicates that photons from the entire absorbed wavelength range can contribute to photogeneration of electron/hole pairs and separation and collection of an appreciable number of them. The low external quantum efficiency however, suggests that the majority of excitons are formed outside the space charge region, in the bulk of the material, and as such recombine. This effect is likely caused by the densely-packed structure, as the distance excitons must diffuse to reach extraction is much greater than in mesoporous or nanostructured materials (see Fig. S5). The presence of many defects within the crystal structure is also a factor that can slow the diffusion of photogenerated charges, increasing recombination and decreasing the quantum efficiency.

The low porosity of our nitride thin films may be caused by the long deposition times or by the growth mechanism. Formation of the thin films by AACVD is expected to occur through two mechanisms, either heterogeneous or homogeneous reactions of the precursor droplets^38^. Homogeneous reactions occur when the solvent in the aerosol droplet evaporates first, facilitating the reaction of the precursor compounds in the gas phase. This tends to form thin films with a highly nanostructured, mesoporous or hierarchical structure^38^. Conversely heterogeneous reactions occur on the heated substrate, facilitating slower evaporation of the solvent, leading to a more compact morphology. Horizontal AACVD, whereby the precursor aerosol enters the heating chamber horizontally and moves across the heated substrate, is known to favor heterogeneous reactions. Evaluation of the cross-sectional SEM images for different thickness films, see Supporting Information Fig. S5, shows that even films deposited for 20 mins have a densely-packed morphology. This supports the proposition that the horizontal aerosol flow is the cause for the more compact nature of the thin film and that is it not predominantly caused by lengthy deposition times. The low photocurrent could be caused by poor crystallinity of the films and their very low porosity, however, enhancements of this porosity could lead to a very significant increase in the resulting quantum efficiency and photocurrent output. A particular advantage of the AACVD methodology is that through modifying appropriate deposition parameters/conditions, one can shift the balance between the heterogeneous and homogeneous reactions. Altering the solvent, temperature, precursor flow rates and aerosol direction can all lead to changes in the ratio of hetero and homogeneous reactions, leading to dramatic changes in the morphology, as previously shown in work by us^38,39^. Efforts are currently ongoing into the modification of the nitride thin film morphologies through manipulation of the AACVD deposition parameters but is currently beyond the scope of this work.

A noteworthy feature of the nitride thin films is the dramatic difference in electrochemical activity with the apparent change in the dominant crystallography. The low indium content composites exhibit the dark current onset, signifying the electrolysis of water, at around 1.7 V vs Ag/AgCl. Conversely the high indium content films with the InN dominant structure, show dark current onset in the 1–1.2 V vs Ag/AgCl range in the 1 M H_2_SO_4_ electrolyte solution. A shift in dark current onset to lower applied potentials is usually indicative of an electrocatalytic process. To ensure that our measurements are not interfered by the oxygen reduction reaction (ORR), current-voltage plots of InN thin films were measured in a deoxygenated electrolyte solution under the presence of argon. No difference was seen in this response, indicating the ORR was not taking place. Additionally, careful inspection of the dark current response throughout the measured region indicates that there are no other redox reactions taking place in the applied potential window.

In order to monitor the stability of the nitride thin films, which is another potential cause for a rapid increase in dark current, current density vs time plots were recorded under 1 Sun illumination in 1 M H_2_SO_4_ buffered by the pH 7.4 Potassium Phosphate (KPi) solution at 1.23 V vs RHE. The stability for the 11% indium thin film is representative for the composite thin films and is shown in Fig. 6D. The stability of the nitride thin films was found to be excellent, with little in the way of photocurrent degradation during the 1 hour measurement time. The photocurrent for InN is also stable over the 1 hour measurement time in both acid and neutral (see Supporting Information Fig. S9), which suggests that the sharp dark current onset is an electrocatalytic process, and not due to other redox reactions or degradation of the thin films. Photodegradation often plagues many metal oxide semiconductor materials but is clearly not an issue for the polycrystalline nitride materials under these conditions^77,78^. Furthermore most photoanodes used for photoelectrochemical processes are not stable in acid and only of limited stability in neutral solutions. The fabrication of acid and neutral stable photoanodes, such as our polycrystalline InN, GaN and their composite thin films, are highly sought after for many photoelectrochemical, solar cell and thermoelectric devices^79^.

## Conclusions

In this study, we have demonstrated that polycrystalline InN, GaN and systematically controlled In_x_Ga_1−x_N composite thin films can be successfully fabricated on fluorine-doped tin oxide coated glass substrates by AACVD, which is a flexible and scalable technique. Variation in the indium content of polycrystalline nitride composite thin films was found to lead to a dramatic shift in the optical absorbance. Importantly, the band edges of the composites shift between that of GaN to that of InN, which is a fundamental objective of many studies reported in the literature to date. The trend between the photocurrent onset potential of the polycrystalline InN, GaN and composites and the band edges determined by Mott-Schottky analysis correlates very well. The 50% composite thin film had an 8% external quantum efficiency. Photocurrent density is relatively low, which is attributed to the high packing density of films and their low surface area, but the stability of the composite nitride thin film (which corresponds 11% indium) and InN film were found to be excellent, with little in the way of photocurrent degradation during the 1 hour measurement time. We believe that these findings will have an immediate and significant impact on the current efforts on low-cost fabrication of bandgap tunable polycrystalline III-nitride semiconductor thin films for wide range of applications.

## Supplementary information

Supplementary Information

## Data Availability

The datasets generated during and/or analysed during the current study are available from the corresponding author on reasonable request.
