# Supplementary material for: Low-cost Fabrication of Tunable Band Gap Composite Indium and Gallium Nitrides
Source: Sci Rep. 2019 Feb 19;9:2313. doi: 10.1038/s41598-019-38882-3 (PMC6381210; doi:10.1038/s41598-019-38882-3)
Supplement: Supplementary file 1 — Supplementary Information [file 41598_2019_38882_MOESM1_ESM.docx]

Supporting Information for the Manuscript “Low-cost Fabrication of Tuneable Band Gap Composite Indium and Gallium Nitrides”

*Andrew McInnes^1^, Jagdeep S. Sagu^1^*, Diana Mehta^1^, K. G. Upul Wijayantha^1^**

^1^Energy Research Laboratory, Department of Chemistry, Loughborough University, Loughborough, LE11 3TU

Corresponding Author: Dr. Jagdeep S. Sagu

Email: J.S.Sagu@lboro.ac.uk

Phone Number: +44 (0)1509 564129

Corresponding Author: Prof. K. G. Upul Wijayantha

Email: U.Wijayantha@lboro.ac.uk

Phone Number: +44 (0)1509 222574

Figure S1 - Schematic of Horizontal AACVD with dual precursor inputs


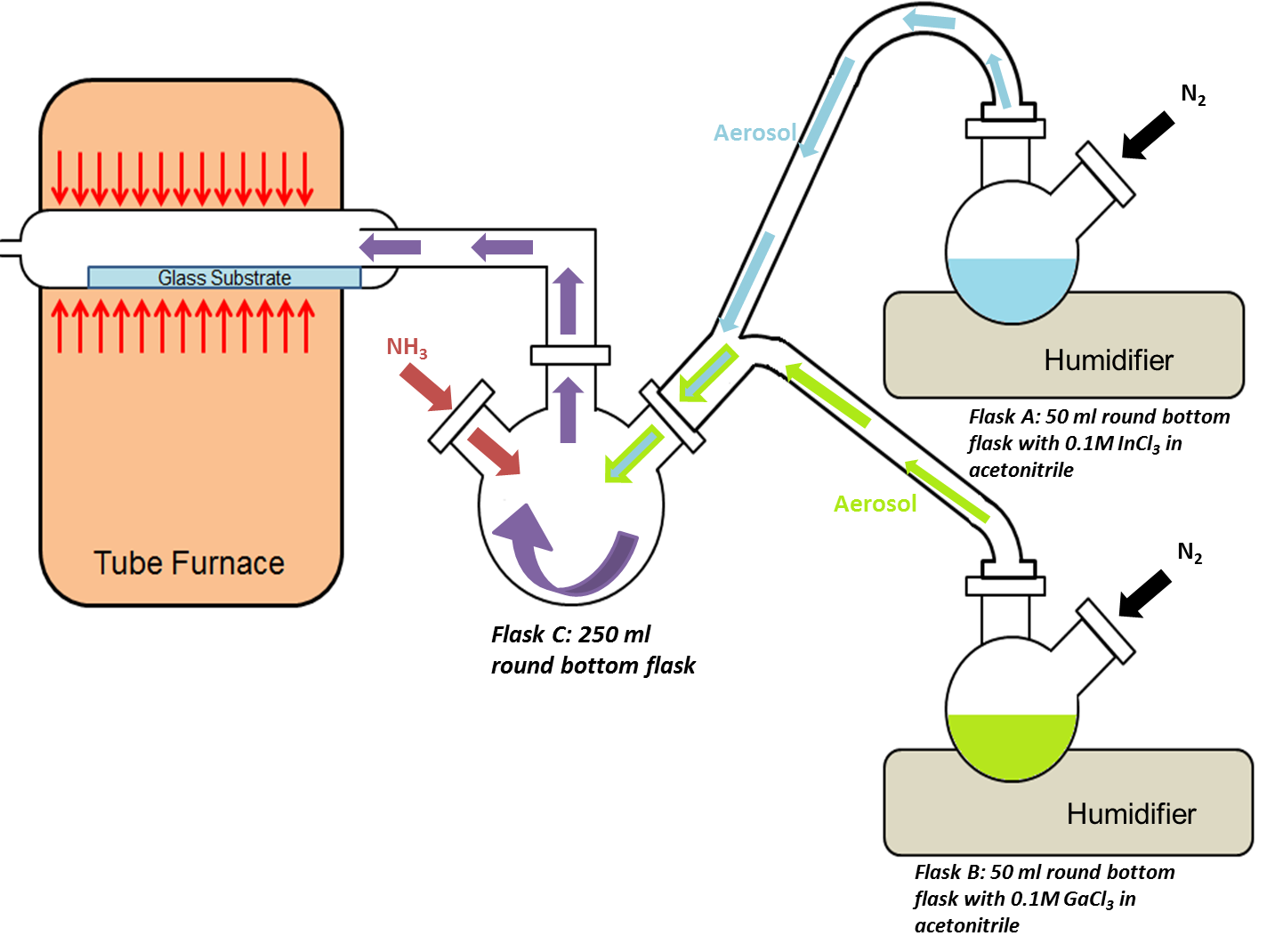


Figure S2 - EDX plots of thin films fabricated with indium precursor flow rate ratios of 100% / InN (A), 89% (B), 65% (C), 50% (D), 35% (E), 11% (F), 0% / GaN (G)


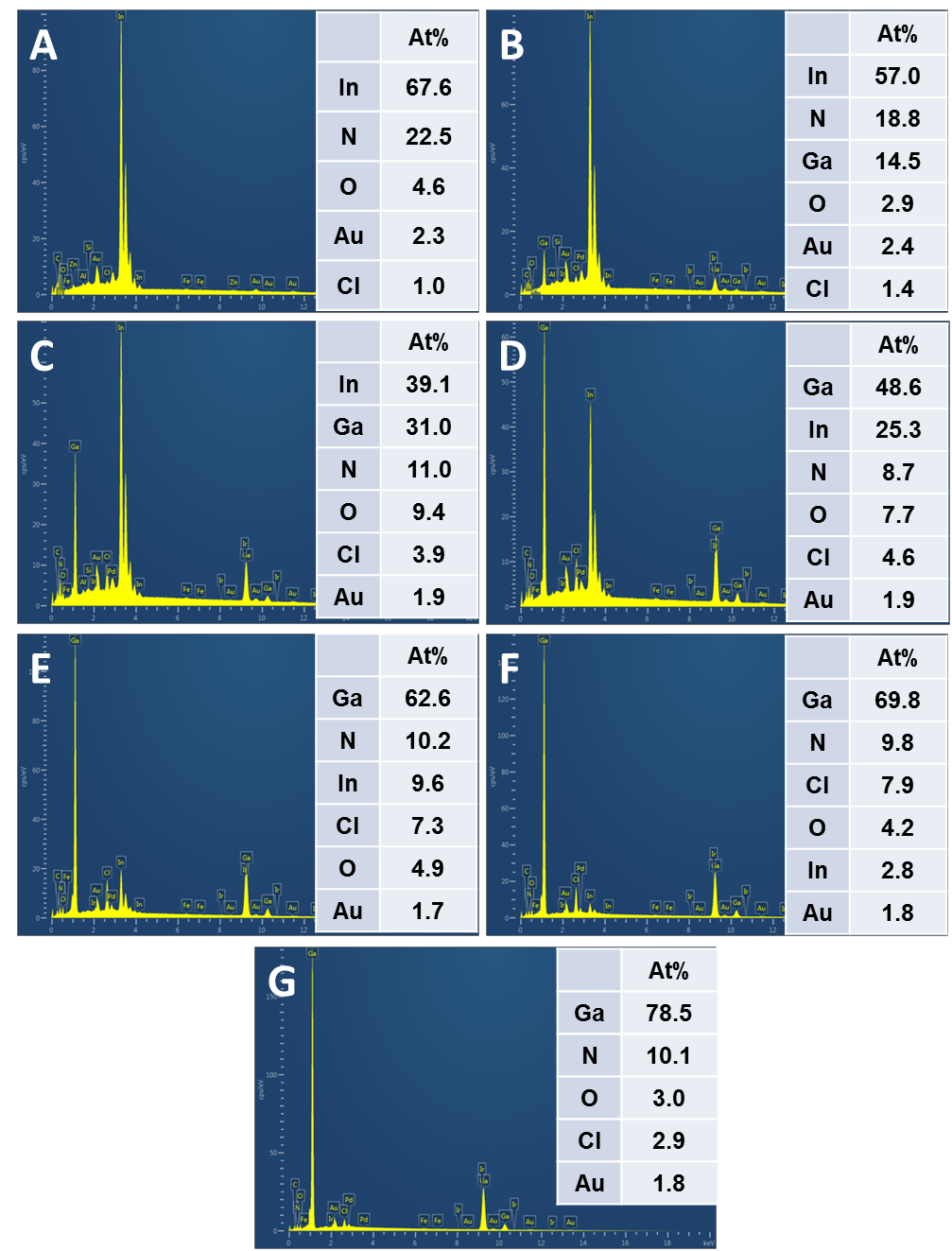


Figure S3 – (ROTATED + cont next page) XPS plots for the range of composite nitride thin films studied. Plots for nitrogen 1s peaks (top row), Ga 3d peaks (middle row) and In 3d peaks (bottom row) are shown for each composition film respectively.


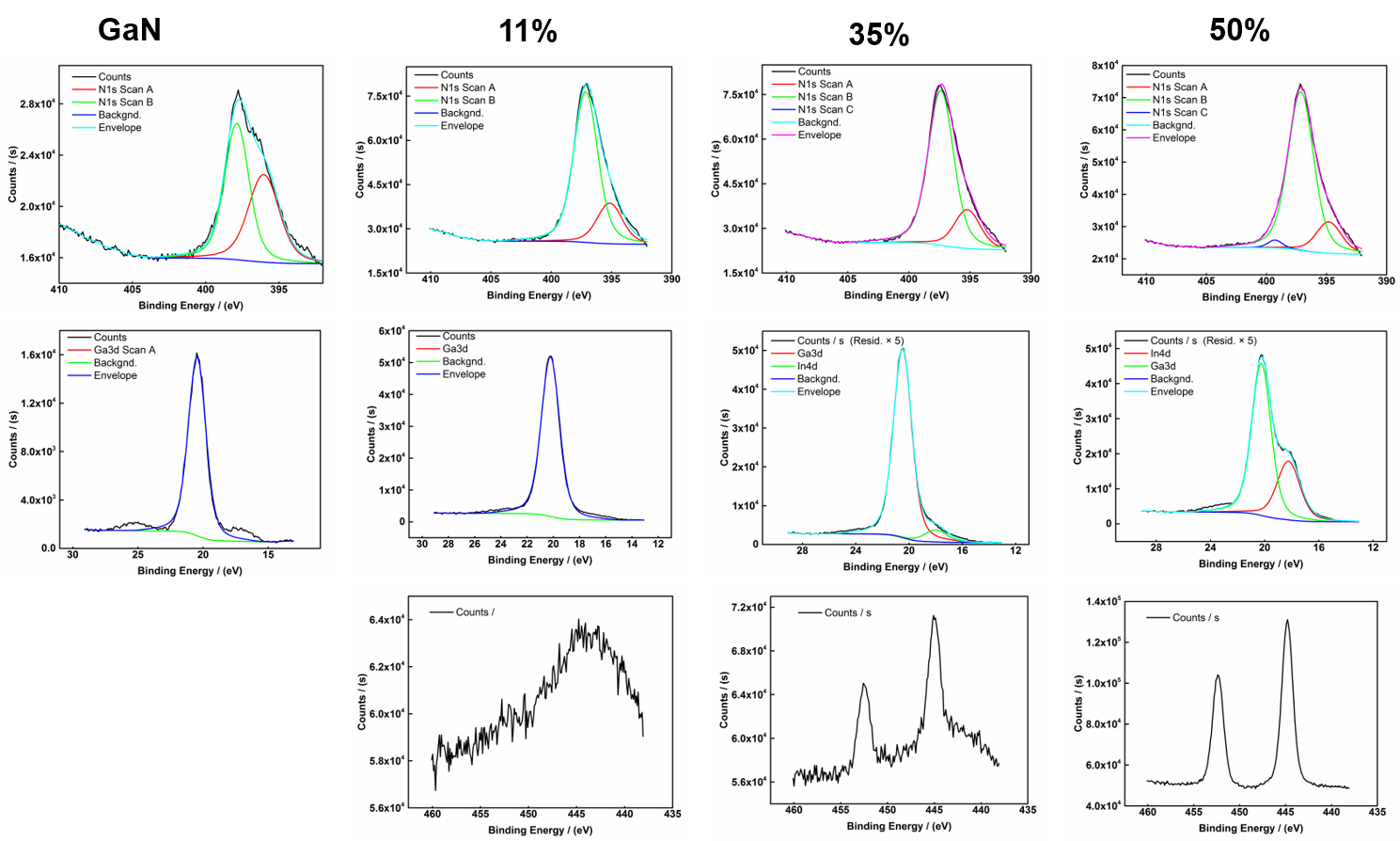


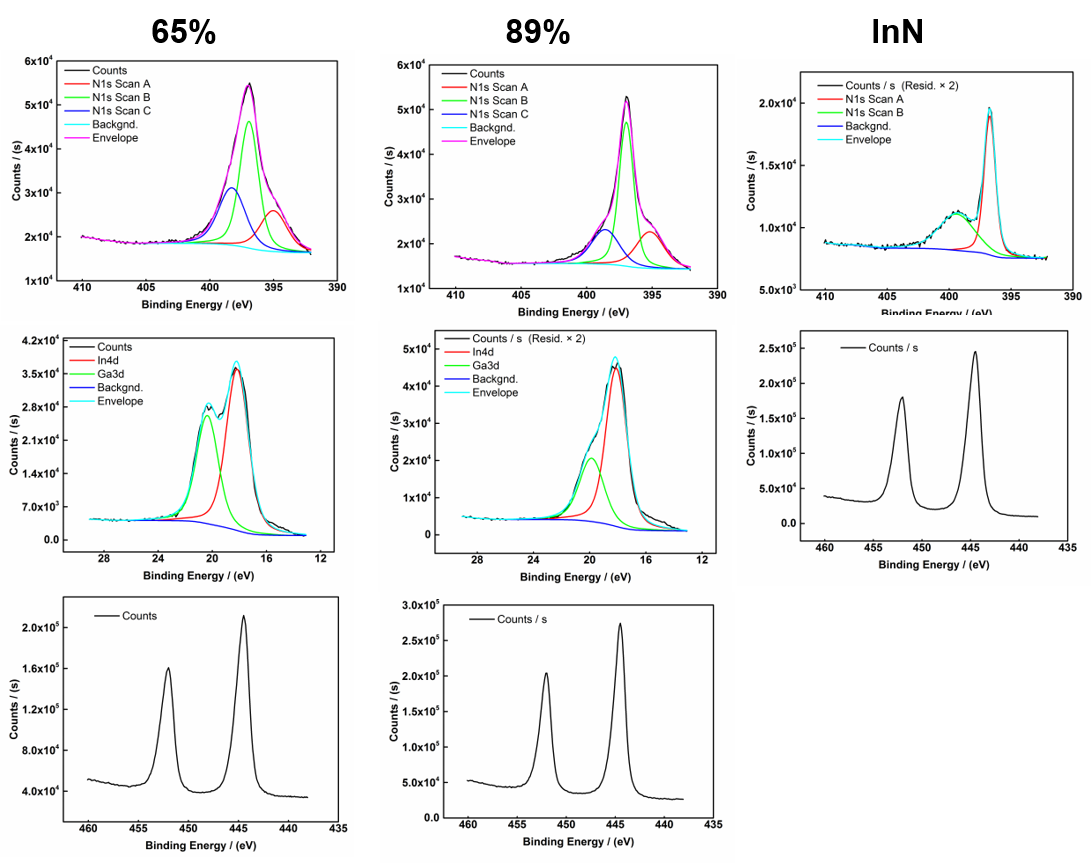


Figure S4 – A) Thickness of thin films with respect to the deposition time. Error bars are plotted from the standard deviation of deposited thin films; B) band gap vs the thickness of thin films; C) average band gap vs the time with error bars due to standard deviation of data points.





Figure S5 – Cross-sectional SEM images of composite nitride thin films deposited for 60 mins, 50 mins, 40 mins, 30 mins and 20 mins respectively. ImageJ was used to measure the thickness of the film which is approximately to 1 decimal place.


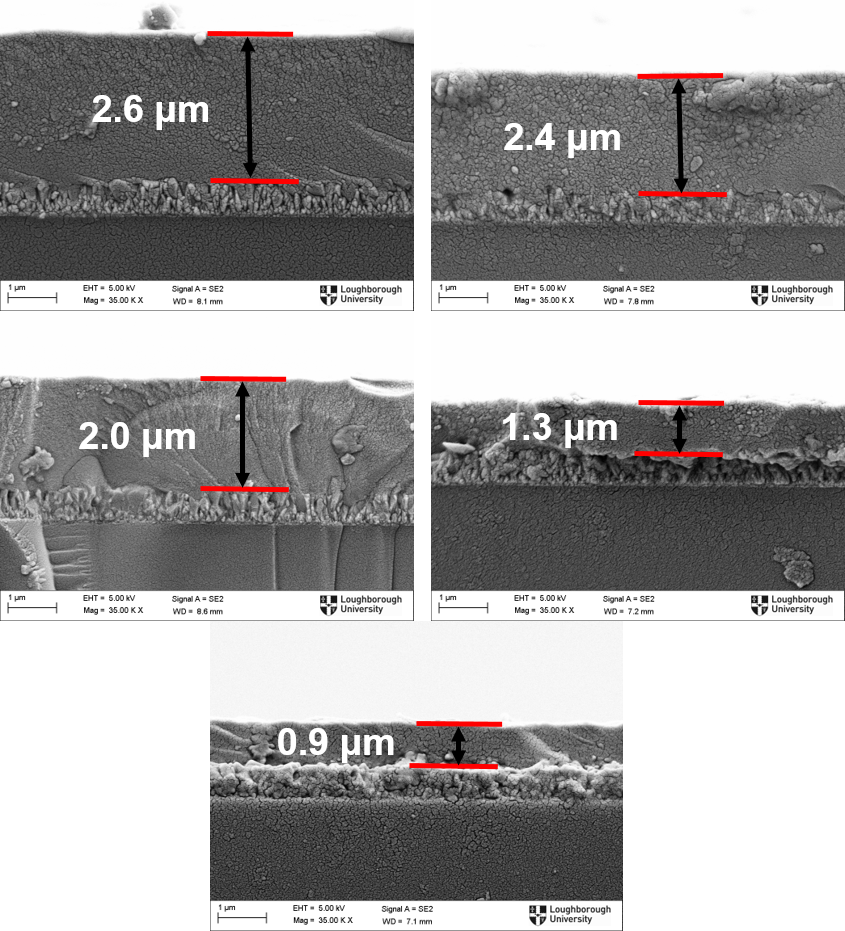


Figure S6 - SEM images for the same composite nitride thin films as shown in figure S5 fabricated with deposition times of 60 mins (A), 50 mins (B), 40 mins (C), 30 mins (D) and 20 mins (E)


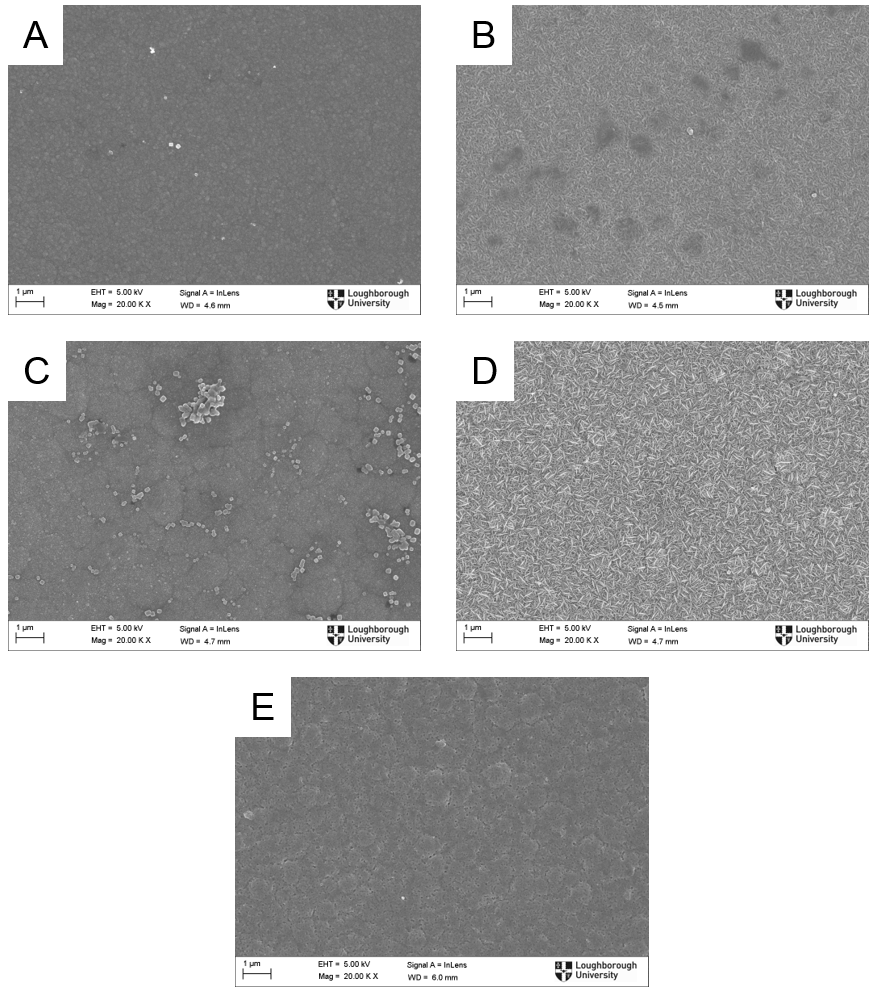


Figure S7 - EDX plots for thin films fabricated for 60 mins (A), 50 mins (B), 40 mins (C), 30 mins (D) and 20 mins (E)


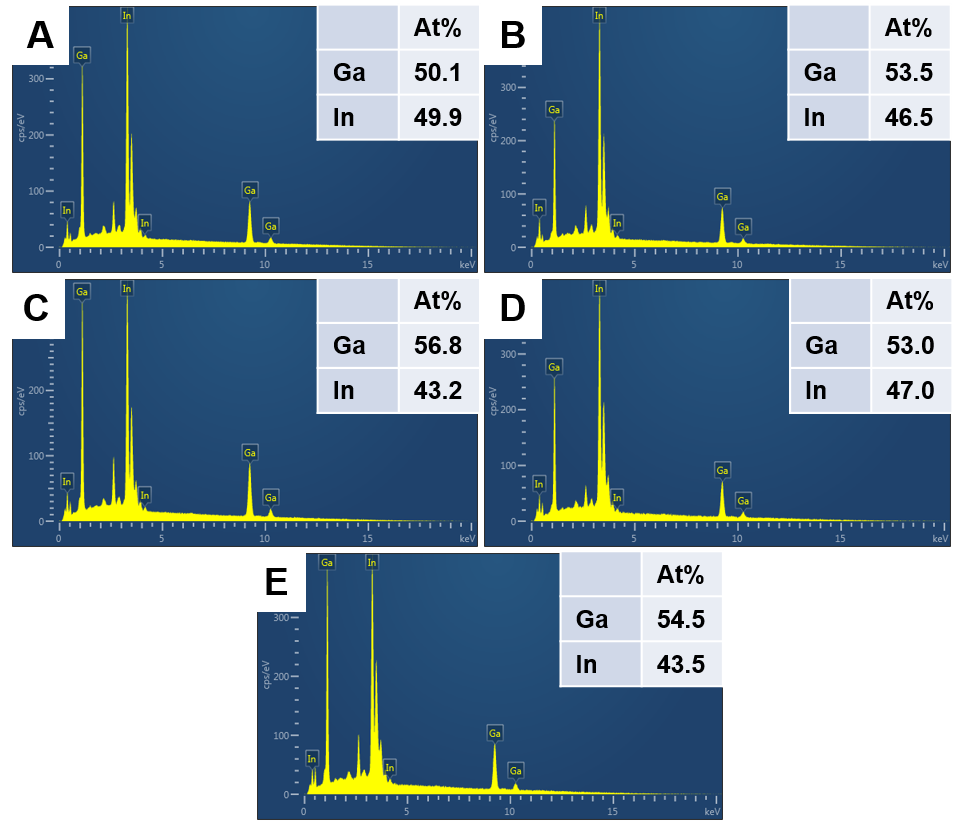


Figure S8 – Chopped Current Density vs Voltage plots for the nitride thin films measured in 1M sulfuric acid for composite thin film nitrides with 0% / GaN (Black), 11% (Red), 35% (Green), 50% (Blue), 65% (Cyan), 89% (Pink) and 100% / InN (Purple) indium percentage by precursor flow rate. Here we observed higher photocurrent for the 11 and 35% thin films, undoubtedly due to different porosity between thin films.





Figure S9 – Photocurrent vs time for InN thin films in 1M H_2_SO_4_ (Black) and in pH 7.4 Potassium Phosphate buffer (Red). The plot shows good stability for the InN albeit with low photocurrent.





S10 Tauc plots for the 50% In_x_Ga_1-x_N composite showing an absorption onset of 2.34 eV, which is in extremely close agreement to the photocurrent onset given by the IQE





Figure S11 – Mott-Schottky plots for composite thin film nitrides with 100% / InN (A), 88% (B), 65% (C), 50% (D), 35% (E), 11% (F), 0% / GaN (G) indium percentage by precursor flow rate.

|  |  |
| --- | --- |
|  |  |
|  |  |
|  | |

S12 Dark current linear sweep voltammogram measured in 1M sulfuric acid for composite thin film nitrides with 0% / GaN (Black), 11% (Red), 35% (Green), 50% (Blue), 65% (Cyan), 89% (Pink) and 100% / InN (Purple) indium percentage by precursor flow rate. Here we can see the current increase, due to the electrocatalytic splitting of water, for each composite. The peak in the InN response is likely due to a redox active impurity on the surface, which could be the metallic indium or indium oxide.
